# The Family Dentist

**Published:** 1914-09-15

**Authors:** A. E. Webster

**Affiliations:** Toronto, Canada


### THE FAMILY DENTIST.

BY A. E. WEBSTER, MD., D.D.S., TORONTO, CANADA.

The family physician is passing in the larger centers of population. The family dentist is in a fair way to follow. In an address before the Academy of Medicine, Toronto, Dr. Graham Chambers pointed to some of the reasons for the physician losing his position as the family adviser. Chief among these reasons was the physician's failure to act as a teacher of personal, family and public health. If the family physician had done his duty as a teacher public health officers would never have been needed. The tendency today is towards making all physicians public servants and not family physicians. The Canadian physician does not take kindly to this change yet he encourages it by asking for public aid for instructing people in matters of health. Another reason for the change is the development of the specialist. Patients often rush from one specialist to another to their disadvantage.

These very things are happening in dentistry. Public dental service has taken hold of the people. Dentists are being employed to teach the public how to avoid diseases caused by the teeth. Public dental clinics are organized as state institutions. Dentists are employed by the year to care for the teeth of the poor.

Specialists are found in every city. Dentists in smaller places are sending patients to others of wider experience. All of this and other things are tending to weaken the place of the family dentist. A strong factor in the change was the introduction of business methods in dental practice A few years ago dental magazines were filled with articles on how to conduct a dental practice. The discussion of the subject has been a great benefit to individual dentists. It has awakened in them an appreciation of the value of their own time and also that of others. More dentists are now being paid for skill and service than ever before. Unfortunately there are many advocates of a time and material basis of charge.

It is the method of bargaining or contracting concerning dental operations or dental service so strongly advocated by itinerant specialists going from city to city giving special courses on business methods for large fees that is fatal to the family dentist. The old school discouraged the bargaining patient, the new school teaches bargaining about everything. A family practice cannot be developed nor can the best be done for the patient when each dental service must be the subject of a contract. To apply the new method to an established family practice must necessarily mean not only the change in the business relations but also a change in the methods of giving service. For example: A child of twelve who has been under a dentist's care since babyhood calls at his office and needs prophylaxis or an amalgam filling. The dentist who has just paid fifty dollars for instruction in how to conduct a dental practice says to the child the fee for the hour I will give you will be five dollars, half of which must be paid before the operation begins and the balance when completed, or since it can be completed in one sitting I will expect the whole amount. The child brings this five dollars to pay for the operation. The traditional family dentist loses some of his grip at once. Like the thrifty house wife who pays cash for her groceries. She gets butter from one store, fruit from another, because she has learned she gets better value for her money. So with the child who has five dollars in his pocket to spend on dentistry he starts out to bargain with dentists because he knows that is the way it is done. Naturally if he can get an hour's work from another dentist for four dollars he is in one dollar to the good. The housewife saw the butter, the fruit, and the potatoes, and could judge of their value, but the lad who bargained for his dentistry was not able to judge of its value at the time or perhaps could never know. If, the housewife got tasteless fruit she lost only what she paid in excess of its value, if the boy got poor dentistry he not only lost what he paid for it, but perhaps lost a tooth or all the teeth he had and his good appearance and health into the bargain.

By the bargain method the dentist does what he contracted to do and then the deal is closed. He makes a bridge for fifty dollars, but since he finds a cavity in an adjoining tooth he must not touch it until it is contracted for and the cash laid down. If the practice of dentistry were making individual bridges or crowns or fillings for people who applied for them then the contract method of practice would be best for the public. Dentistry practiced in the best interests of the patient must have a broader outlook than single bridges, crowns or fillings. Many a good-looking filling or crown has caused years of ill health and suffering. The real practice of dentistry must have for its keynote *prevention* otherwise it is a failure and the state must step in. There can be no personal and vital interest between the dentist and his patient if there is such a mistrust of honesty that each individual service must be contracted for before it is begun.

The specialist in dentistry tends to destroy family practice and he only exists because of the incompetency of dentists. Specialists as a rule treat only chronic cases and chronic cases would rarely exist if their causes had been recognized. The general practitioner fails in his duty who allows pyorrhea, irregularities of the teeth, or chronic abscesses to develop.

The dental society is one of the strongest factors in destroying the position of the family dentist. Every department of dentistry must be presented by a specialist, who is trained in treating chronic diseases which should never have been allowed to begin. The ordinary practitioner in whose practice these cases begin is not allowed to say a word. The crown and bridge and partial denture *artist* goes before a society and describes the treatment of cases which should never develop in a family practice. Such cases develop mainly where each sitting closes the contract.

The operative *artist* describes how he makes out a chart of the cavities and then arranges the necessary appointments, and gets a retainer fee. At each subsequent sitting the chart is brought out to work from. In a properly conducted family practice several cavities could hardly occur between visits. The specialists in business methods of conducting a dental practice talks about deposists of fifty dollars, a hundred dollars, or perhaps five hundred dollars. A large fee at any one time in a family practice is an indication of previous neglect or bad judgment on the part of the dentist. If the business specialist would devote attention to how dentists should save and judiciously invest and spend what they receive it would be quite as much to the point as spending thought on how to get it from the people.—*Dominion Journal.*

